# National Clinical Skills Competition: an effective simulation-based method to improve undergraduate medical education in China

**DOI:** 10.3402/meo.v21.29889

**Published:** 2016-02-16

**Authors:** Guanchao Jiang, Hong Chen, Qiming Wang, Baorong Chi, Qingnan He, Haipeng Xiao, Qinghuan Zhou, Jing Liu, Shan Wang

**Affiliations:** 1 Educational Department, Peking University People's Hospital, Beijing, China; 2 Peking University People's Hospital, Beijing, China; 3 Ministry of Education, People's Republic of China, Beijing, China; 4 Norman Bethune Health Science Center, Jilin University, Jilin, China; 5 Xiangya School of Medicine, Central South University, Changsha, China; 6 Zhongshan School of Medicine, Sun Yat-Sen University, Guangzhou, China

**Keywords:** clinical skills, competition, simulation-based medical education, undergraduate medical education

## Abstract

**Background:**

The National Clinical Skills Competition has been held in China for 5 consecutive years since 2010 to promote undergraduate education reform and improve the teaching quality. The effects of the simulation-based competition will be analyzed in this study.

**Methods:**

Participation in the competitions and the compilation of the questions used in the competition finals are summarized, and the influence and guidance quality are further analyzed. Through the nationwide distribution of questionnaires in medical colleges, the effects of the simulation-based competition on promoting undergraduate medical education reform were evaluated.

**Results:**

The results show that approximately 450 students from more than 110 colleges (accounting for 81% of colleges providing undergraduate clinical medical education in China) participated in the competition each year. The knowledge, skills, and attitudes were comprehensively evaluated by simulation-based assessment. Eight hundred and eighty copies of the questionnaires were distributed to 110 participating medical schools in 2015. In total, 752 valid responses were received across 95 schools. The majority of the interviewees agreed or strongly agreed that competition promoted the adoption of advanced educational principles (76.8%), updated the curriculum model and instructional methods (79.8%), strengthened faculty development (84.0%), improved educational resources (82.1%), and benefited all students (53.4%).

**Conclusions:**

The National Clinical Skills Competition is widely accepted in China. It has effectively promoted the reform and development of undergraduate medical education in China.

Since the twenty-first century, due to the rapid development of medical education and constant evolution of educational philosophy and methods, there has been an international trend in the adoption of competency-based medical education in undergraduate medical education (1). For a long time, Chinese undergraduate medical education programs placed too much emphasis on knowledge and insufficient emphasis on skills, attitudes, and their synthesis into observable competencies (2). Facing a new trend in medical education and to meet the needs of patients and social development, the Chinese government issued several documents to promote medical education reforms that aim to strengthen the clinical competency and professionalism of medical students. These reforms such as ‘The standards of undergraduate medical education in China’ (3) and ‘The implementation of distinguished doctor education and training plan’ (4) were issued by the Ministry of Education and the Ministry of Health in 2008 and 2012, respectively.

However, China has a vast and complex system of health professional education. Approximately 67,000 students graduate with a bachelor's degree in medicine from 136 medical colleges in China each year. The broad area and large disparity in regional economic development in China has resulted in many problems including the unbalanced distribution of teaching resources, disparity in teacher's expertise, differences in the understanding of modern teaching methods, failure to cultivate the clinical skills and competence of medical students, and adaptability to new concepts of medical education in these medical colleges (5). Moreover, there is no effective platform available for universities to exchange experiences with each other. How to guide medical colleges to carry out reform and improve the quality of education is a major issue faced by Chinese medical educators. The Research Center of Clinical Medical Education of the Ministry of Education (hereafter referred to as the Center) was subordinated to the Chinese government with an aim of transmitting new teaching concepts among Chinese universities, promoting the reform of medical education, and providing an exchange platform for Chinese universities. Therefore, entrusted by the Ministry of Education, the Center used the National Clinical Skills Competition (hereafter referred to as the competition) as a method of advocating advanced educational philosophy and methodology, and as a platform for exchanging experiences between universities.

With the spirit of ‘show style, uphold learning, refine skills, and honor professionalism’, the competition has been held for 5 consecutive years since 2010. The current study summarizes the participation and the questions in the finals of the competition, as well as an evaluation of the competition by teachers and students, and analyzes the guiding and promoting role of the competition in undergraduate medical education reform, providing new ideas for future development of the competition, as well as referential experiences for medical education in other countries.

## Methods

### Organization of the competition

**Fig. 1:**
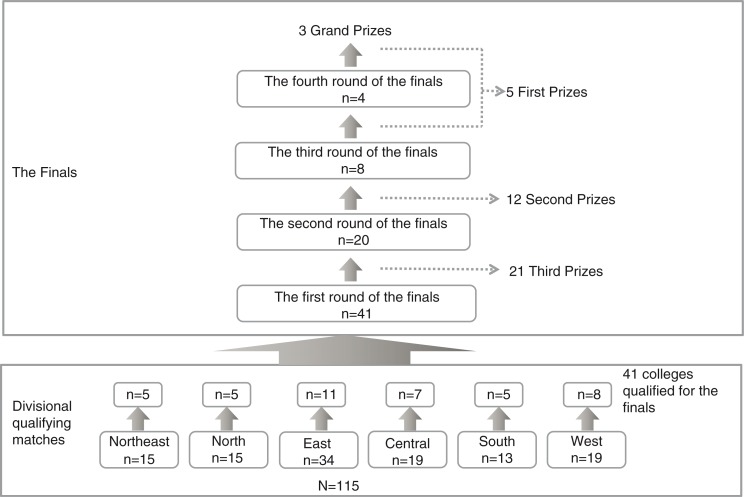
Flow chart of the 5th National Clinical Skills Competition 2014.

The competitions were sponsored by the Center and hosted by different colleges chosen through review. The competition was divided into the divisional qualifying matches and the final. First, the country was divided into six areas according to the administrative regions of China, including the northeast, north, east, central, south, and west China, with 10–40 medical colleges in each division. Each division organized the divisional qualifying match in April each year, with about 36% of top colleges entering the national final. In the final held in May each year, the college rankings were determined through 3–4 rounds of competition (Fig. 1).

### Participants

The participants were selected from undergraduate medical students at the stage of clinical practicum (usually at the fifth year or equivalent). Each college had a team of four excellent students who were selected by competing at the school level to participate in the competition and represent the school. These students completed the mission of the competition collectively or individually as required. Competition awards are conferred on the students as well as the school.

### Competition design

The Center announced the scope of the annual competition. Under the supervision and guidance of the Center, the competition hosts organized their own experts to design the competition questions and the scoring criteria. Based on the simulated scenario and clinical cases, the competition highlighted the evaluation of knowledge, skills, and attitudes.

### Competition rules

There were two forms of competition: objective structured clinical examination (OSCE) and the ‘track contest’. OSCE was mainly used in the divisional qualifying matches. Track contest was similar to the 100-m race, with 3–6 tracks in parallel and 4–6 test stations on each track. Each test station simulated the clinical scenario through task trainers, high fidelity simulators, and standardized patients, assessing one or more skills. Each track accommodated one team with three players (and one alternate), who finished the examination content individually or cooperatively as a team in accordance with the requirements. Each team started from the first station of each track at the same time and passed through all the reference stations sequentially. Proficiency of completion was also evaluated when assessing the skills and quality of completion. Track contest was mainly used in the finals with a much stronger competing atmosphere.

### Competition referees

The Center organized experienced senior clinical doctors with related professions from the hosting colleges and other non-participating colleges to serve as referees. There were at least two referees who scored each skill, and the average of their scores was used. The assessment scale has been evaluated by inter-rater reliability with high objectivity and feasibility. The referees received training before the competition. The competition was double-blind—neither the referees nor the students knew each other. Training will be offered to judges before the competition, through which the judges will be informed of the rules, procedures, ways to use evaluation forms, scoring standards, and tactics dealing with emergencies. The judges will arrive at the venue and conclude the requirements prior to each round of the competition, so as to allow sufficient time to prepare. The individuals whom set examination questions will help the judges familiarize themselves with simulators, scoring standards, the dos and do nots, and grading techniques responding to different situations.

### Analysis of the effectiveness

Using a summary of the participation and questions of each competition, the influence and guidance quality of the competition were analyzed. By distributing questionnaires to all the participating colleges, the effects of the competition in promoting undergraduate medical education reform such as updating the curriculum model and instructional methods, strengthening the training of teachers, improving educational resources, and benefiting the students were evaluated.

### Statistical analysis

Descriptive statistics were generated for all data.

## Results

### Participation in the competition

**Fig. 2:**
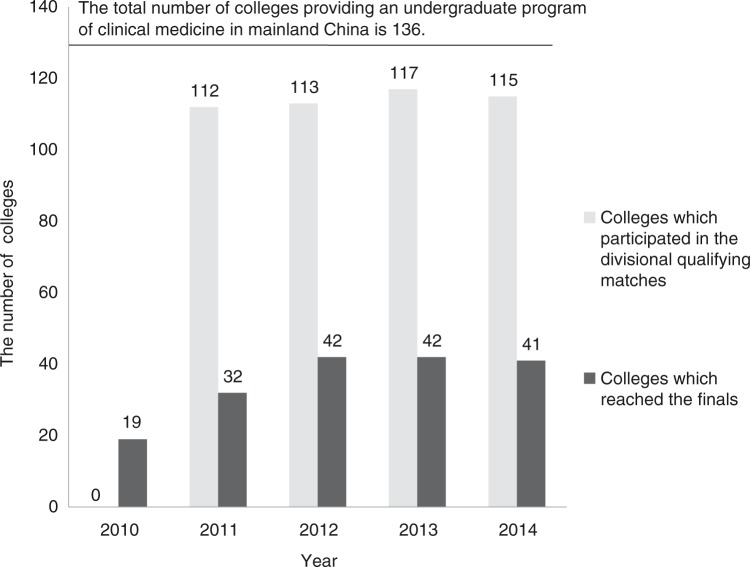
The number of colleges that participated in the competition each year.

The competition has been held five times since 2010 with a gradual increase in the number of participating colleges. Currently, there are 136 colleges providing undergraduate program of clinical medicine in mainland China, more than 81% of which have participated in the last four competitions (Fig. 2). The competition involved medical students, teachers, teaching administrative personnel, and related policy makers in the government. Each year, every college selects four students through a competitive process at school level as their representatives, with a total of approximately 450 students from more than 110 colleges directly participating in the competition. However, the estimated number of students participating at school-level selection was more than 20,000. Approximately 27 teachers in each college (about 3,000 teachers nationwide) participated in pre-competition training activities. Approximately 1,000 teachers participated in designing and refereeing work at the six divisional qualifying matches and the finals.

### Contents of the competition

**Fig. 3:**
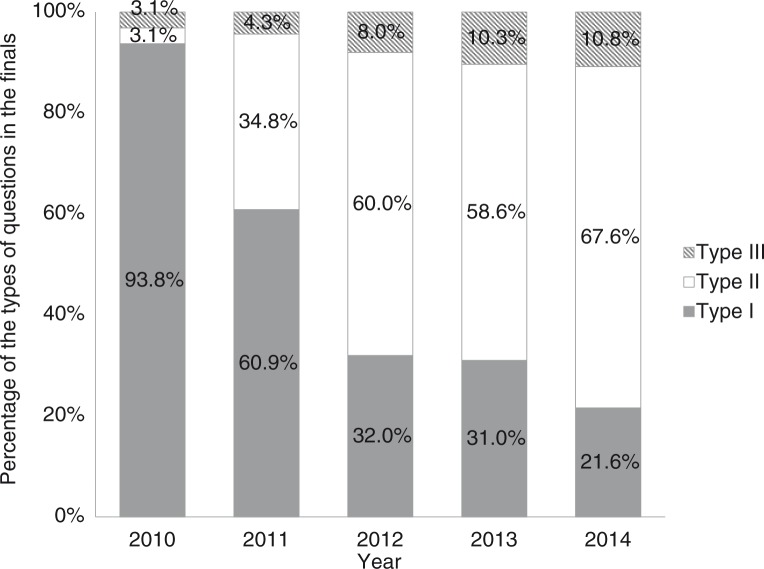
The variation of the questions in each final competition.

The competition mainly covered internal medicine, surgery, gynecology and obstetrics, pediatrics, ophthalmology, ENT, and dermatology. The scope of the competition covered approximately 100 clinical skills (Table 1). According to the different requirements of the assessment, the questions in the finals can be divided into three categories: type I, using task trainers to evaluate the individual technical skills, which focused on the proficiency and quality of the performance; type II, using SP or task trainer associated with short clinical cases to assess technical skills, clinical reasoning, and professionalism; type III, integrates SP, high fidelity simulator or other clinical simulations, and long clinical cases, to evaluate the comprehensive ability and professionalism of students. The proportions of the three types of questions in the finals continuously evolved. The first and second competitions mainly used type I/II questions, while the remaining competitions mainly used type II/III questions (Fig. 3).

**Table 1:** The clinical skills assessed in each final competition

	Final competition				
	
Competence assessed in the competition	1st	2nd	3rd	4th	5th
Knowledge	•	•	•	•	•
Technical skills					
Physical examination and history taking		•	•	•	•
ECG and X-ray reading	•	•	•	•	•
Aseptic techniques	•	•	•	•	•
Surgical skills (incision, suture, knot, stitches taking out, dressing change)	•	•	•	•	•
Surgical skills (debridement, resection of body mass, incision, and drainage of surface abscess)		•	•	•	•
Bone fracture fixation		•	•	•	•
BLS/ACLS (CPR, endotracheal intubation, electric defibrillation)	•	•	•	•	•
Thoracocentesis, peritoneocentesis, lumber puncture, bone marrow puncture	•	•	•	•	•
Venipuncture, arteriopuncture	•	•	•	•	•
Nursing skills (nasogastric tube, sputum suctioning, urethral catheterization and enema)	•	•	•	•	•
Injection skills (intramuscular, intracutaneous, subcutaneous)	•	•	•	•	•
Don and remove isolation gown, needle injury treatment	•		•		
Gynecological skills (pelvic examination and external pelvimetry, cervical cytological exam)	•	•	•	•	•
Obstetrics skills (four maneuvers of leopold, digital examination per rectum and vagina, pregnogram study, partogram study, fetal heart monitoring)	•	•	•	•	•
Neonatal nursing (neonatal measurement, formula preparation, scalp vein-puncture, pediatric bone marrow puncture)	•	•	•	•	•
Examination skills in ophthalmology, ENT, and dermatology		•	•	•	•
Trauma management (handling patients with a variety of trauma conditions including spinal injury)				•	•
Fundamental laparoscopic surgery					•
Central vein catheter, chest tube removal, and vesicopuncture		•	•	•	•
Non-technical skills					
Communication, teamwork, leadership, decision-making, and situation awareness	•	•	•	•	•
Critical thinking			•	•	•
Professionalism and ethics					
Respect, compassion for the patient and commitment to patient confidentiality	•	•	•	•	•
Laws and regulations		•	•	•	•

### Evaluation of the effects of the competition

Eight hundred and eighty 5-point Likert scale questionnaires were distributed to 110 participating colleges and the interviewees including teachers, administrative staff, and students who did or did not participate in the competition. Seven hundred and fifty-two usable questionnaires were recovered from 95 colleges (including 185 teachers, 188 administrative personnel, 189 participating students, and 190 non-participating students). The results showed that the competitions promoted undergraduate medical education reform. The majority of interviewees agreed or strongly agreed that the competition promoted the adoption of advanced educational principles (76.8%), updated the curriculum model and instructional methods (79.8%), strengthened the training of teachers (84.0%), improved educational resources (82.1%), and benefited all of the students, including the non-participating students (53.4%) (Table 2).

**Table 2:** Results of the questionnaire on the effects of the competitions on undergraduate medical education

		Responses to the questions (*n*=752)		
		
Questions	Mean(SD)	Strongly agree or agree	Neutral	Disagree or strongly disagree
1. The competition updates the teaching philosophy. (e.g., promoted the transformation from knowledge-based education to the competency-based education).	3.99 (0.86)	578 (76.8%)	136 (18.1%)	38 (5.0%)
2. The competition reflects the problems of daily teaching. (e.g., reflected the insufficiencies on non-technical skills training, lack of professionalism, irregular performance during procedural skills, separation of simulation training and bedside training, and helped to solve this issue).	3.88 (0.85)	582 (77.4%)	121 (16.1%)	49 (6.5%)
3. The competition promotes curriculum reform and updates instructional methods. (e.g., promoted integration of the simulation-based education into the existing curriculum; strengthened the non-technical skills training and professionalism cultivation).	4.05 (0.87)	600 (79.8%)	116 (15.4%)	36 (4.8%)
4. The competition strengthens faculty development. (e.g., promoted the training of teachers, standardized their clinical skills and behaviors, cultivated more dedicated teachers, and strengthened the construction of faculty incentive mechanisms such as bonuses, promotion and award).	4.07 (0.80)	631 (84.0%)	91 (12.1%)	30 (4.0%)
5. The competition improves educational resources. (e.g., expanding or establishing a simulation center, purchasing more equipment, increasing the budget).	4.16 (0.81)	617 (82.1%)	113 (15.0%)	22 (2.9%)
6. The competition benefits all students (including the non-participated students). (e.g., updated curriculum model and instructional methods, improved educational resources were applied in conventional teaching, which benefit all the students).	3.54 (1.11)	402 (53.4%)	225 (29.9%)	125 (16.7%)
7. The competition can improve teaching quality. (e.g., enhanced the enthusiasm of students and promoted the improvement of comprehensive competence of students, such as communication, clinical decision-making, teamwork, and professionalism).	4.14 (0.72)	633 (84.1%)	107 (14.2%)	12 (1.6%)
8. You will support your college to host the competition in future.	3.10 (1.00)	585 (77.8%)	120 (16.0%)	47 (6.3%)
9. You will support your college to participate in the competition in future.	3.24 (0.80)	624 (83.0%)	106 (14.1%)	22 (2.9%)

The 5-point Likert scale questionnaire was as follows: 1, strongly disagree; 2, disagree; 3, neutral; 4, agree; 5, strongly agree.

## Discussion

The competition is different from the general conference or accreditation with its more extensive participation. First, this is reflected by the large number of participants with wide coverage. For each competition, there were more than 110 medical colleges (accounting for more than 81% of the medical colleges nationwide) and approximately 450 students selected from almost 20,000 medical students representing their colleges. More than 1,000 teachers served as competition experts or referees, and nearly 3,000 teachers participated in pre-competition consultation and training of the students. Second, participation time was long. The competition lasted for 4–5 months, and during this period, each college carried out a lot of related preparatory work, including designing the competition, improving simulators, preparing the competition venues, holding school-level contests to select participants, training the participants, participating in the competition, summarizing, and exchanging experiences. The invested resources and personnel were greater than those at general conferences (6). Third, the competition involved many medical education-related personnel including policy makers, administrators, and executors, together with simultaneous comparative observation of the clinical teaching qualities of many institutions. Since different people have different thoughts and gains, not only it is easier to identify problems, it also allows quick feedback for the attention of policy makers, which helps solve the problems. Such influences are not likely to be achieved by general conferences (7).

Why is such a competition so appealing? First, the organizer has great authority. The Chinese education system is marked by strong government ownership and guidance (5). The Research Center of Clinical Medical Education is an academic committee affiliated to the Ministry of Education and is composed of famous medical educators from medical colleges around the country, whose responsibility is to design and lead the development of clinical medical education in China. Second, the competition advocates modern philosophy of medical education and the application of modern instructional methods, reflecting the tendency in medical education development, which provides ideas for the reform and development of clinical medical education in the participating colleges. Third, the competition provides a good communication platform for different medical colleges, which promotes effective communication among colleges and teachers. Fourth, the performance of students in the competition reflects the achievements and weaknesses of daily teaching, identifies deficiencies in teaching, and seeks improvement, which is more intuitive than an academic conference with quicker feedback. Fifth, obtaining a good result in the competition is an honor for the colleges, teachers, and students (6–9).

The goal of clinical medical education is no longer merely to impart knowledge, but has become more extensive. Medical education is now expected to cultivate students’ skills and professionalism (1). Currently, medical schools in China have adopted comprehensive curricular reform, shifting from a knowledge-based education to a competency-based model and moving from traditional practices to compliance with international standards (5, 10). The questionnaire regarding the effects of the competition in the current study showed that most respondents believed that the competition played a positive role in promoting the reform and development of different aspects of clinical medical education (Table 2). The summary reports also confirmed these conclusions (6–9).

First, the competition promoted the adoption of advanced educational principles and updated the curriculum model and instructional methods. It is well known that assessment drives learning (11). Simulation is an effective measure of training and assessment of various abilities of students (12). The competition evaluates the knowledge, skills, and attitudes of the students through various simulation scenarios and emphasizes competency-based learning through using new or revised questions in each final. For example, in the finals, the questions can be divided into three types. Type I, task trainer is used to evaluate the procedural skills. We improved the model to make the evaluation more objective and meet the needs of clinical practice. For instance, the traditional knot test only assesses the speed of knotting objectively, while the quality of the knot is evaluated subjectively (such as whether the original location was maintained and whether excessive traction was used during knotting, and if the knot is tight and effective). To this end, we improved the simulator, which ties the knot around a latex tube connected to a pull sensor. The pull sensor will set off an alarm when excess traction is used, while circulation of water through the latex tube can indicate poor quality of the tied knot. There have been a variety of modified models, which have been rewarded with 33 Chinese patents, and promoted nationwide. Type II, SP or task trainer combined with short clinical cases was used to assess a number of capacities including procedural skills, clinical reasoning, communication skills, and professionalism. The assessment of removing a closed thoracic drainage tube is taken as an example. For a patient with bilateral spontaneous pneumothorax, the indwelling bilateral thoracic closed drainage tube had been in place for 4 days. The participants are asked to judge whether the tube needs to be removed and the appropriate treatment. Students should take the history of the patient, check the chest X-ray, and carry out a physical examination using the simulator and will then make the right judgments, remove the drainage tube with indication, and inform regarding considerations and further treatment. Type II questions expand on the basis of technical skills, assess the various capabilities of the students, and are currently the most important type of questions in the competition. Type III, using hybridized simulation, composed of SP and high fidelity simulators, combined with long clinical cases, is a comprehensive evaluation of the ability of students. For example, the emergency rescue of a patient suffering from poisoning due to organic phosphorus with decreased blood pressure and heart rate. The team of students need to propose a diagnosis and perform a series of treatments, including obtaining a history of the patient, conduct a physical examination, open a vein, take a venous blood sample for examination, perform tracheal and nasogastric intubation, and conduct gastric lavage. In addition, the team is required to communicate with the family, which is a comprehensive and integrated test of a variety of competencies, including technical skills and non-technical skills (such as teamwork, critical thinking, decision-making, communication, and situation awareness). As shown in Fig. 2, questions in the five finals gradually shifted from type I to type II and III, which demonstrated that the competition guided the change of focus in medical education from cultivation of knowledge and procedural skills training to competency-based education.

Second, the competition strengthened faculty development (8). Teachers should not only be equipped with all the skills, but also standardize their behavior in daily teaching and clinical work, and be a role model, which is essential in medical education (13). From another point of view, the competition is not only a test for students, but also a test for teachers. The participating colleges prepared according to the requirements of the competition and the advocated teaching methods. During this process, the teachers accepted the advanced educational philosophy, standardized the skills and behaviors, learned new teaching methods, and improved teaching quality. Through the competition, many colleges cultivated a group of excellent clinical teachers enthusiastic toward teaching.

Third, the competition improved educational resources (9). The colleges took the competition seriously with increased human, financial, and resources support, which was particularly evident in the host colleges. Normally, the divisional qualifying match would cost $200,000–$300,000, while the final would cost $400,000–$500,000. Therefore, many colleges and universities built or expanded their simulation centers for the competition (9). In addition, sales in the main manufacturers of medical simulators in mainland China increased threefold from 2010 to 2014 at least, which indirectly reflected the promoting role of the competition in simulation-based medical education.

Finally, the competition benefited the students (including the non-participating students). The competition resulted in changes in teaching principles, renewal of teaching methods, strengthening of the teaching environment, and improvement in teachers. Although only a few students participated directly, these changes resulting from the competition will be reflected in the daily teaching (7). In addition, the competition promoted the Center to organize national experts to prepare the “Guidelines on basic clinical practices for China Medicos,” which standardized 60 types of commonly used clinical practices necessaryfor medical students with the total circulation nationwide reaching 40,000 copies, which played a positive role in standardizing the basic clinical practices of medical students in China and improving their clinical competence.

Of course, the competition also exposed many problems. Most of the questions in the competition were simulation-based assessments, while bedside clinical training is more crucial for the cultivation of clinical competence and professionalism of medical students. Although the achievements in simulation-based medical education could be transferred to improvements in clinical competence, to a certain extent (14), these would only serve as effective auxiliary measures of bedside clinical training, but would not replace it (15). The main problems faced by the competition are as follows: how to ensure that the simulation training and evaluation method of the competition are close to clinic practice and translated into improvements in clinical competence. In addition, the other key factors that affect the healthy development of the competition included: how to form a long-term mechanism to maintain the enthusiasm of participating medical colleges; how to balance the competition and the routine medical training for the participants; how to promote normal teaching activities to benefit more students and raise the overall quality of medical education.

## Conclusions

In summary, the competition is currently widely recognized in China as an influential and effective method of promoting the reform and development of clinical medical education. There is potential for applying this competition process in other countries, too.
